# Triggers of Inflammatory Heart Disease

**DOI:** 10.3389/fcell.2020.00192

**Published:** 2020-03-24

**Authors:** Ninaad Lasrado, Bharathi Yalaka, Jay Reddy

**Affiliations:** ^1^School of Veterinary Medicine and Biomedical Sciences, University of Nebraska–Lincoln, Lincoln, NE, United States; ^2^Bristol-Myers Squibb – Hopewell, Pennington, NJ, United States

**Keywords:** autoimmune diseases, myocarditis, pericarditis, endocarditis, microbes, inflammatory heart disease, bacteria

## Abstract

Inflammatory heart disease (IHD) is a group of diseases that includes pericarditis, myocarditis, and endocarditis. Although males appear to be more commonly affected than females, IHD can be seen in any age group. While the disease can be self-limiting leading to full recovery, affected individuals can develop chronic disease, suggesting that identification of primary triggers is critical for successful therapies. Adding to this complexity, however, is the fact that IHD can be triggered by a variety of infectious and non-infectious causes that can also occur as secondary events to primary insults. In this review, we discuss the immunological insights into the development of IHD as well as a mechanistic understanding of the disease process in animal models.

## Introduction

The immune system has evolved to fight infections. Upon exposure to microbial infections, the innate immune cells swiftly come to the body’s defense, and, as infections get established, cells of the adaptive immune system respond to prevent future recurrences. Nevertheless, the immune system is not expected to recognize self-tissues as foreign. Should such misdirected responses occur, the autoimmunity that ensues needs to be distinguished from auto inflammatory diseases. However, both are marked by dysregulated and chronic activation of the immune system against self-antigens in the genetically predisposed individuals.

Autoimmunity can occur in any healthy individuals as evidenced by the detection of low levels of autoantibodies (Waldner, 2009; Lleo et al., 2010). Thus, autoimmunity and autoimmune disease should be distinguished clinically in that autoimmune disease occurs only when autoimmunity leads to tissue damage disrupting the functions of affected organs. The National Institutes of Health estimates that approximately 23 million people (up to 8% of the total population) could be affected by some form of approximately 80 known autoimmune diseases (Selgrade et al., 1999). In fact, autoimmune disease represents the fourth largest cause of disability among women in the United States, and is the eighth leading cause of death in women between the ages of 15 and 64 (Fairweather and Rose, 2004; Benros et al., 2014). Additionally, diagnosis of autoimmune diseases may take up to 5 years, and as many as five doctors may see a patient before proper diagnosis is made (Cassell and Rose, 2003). Economically, annual direct health care costs resulting from autoimmune diseases are estimated to be $100 billion (2005). Mechanistically, autoimmune diseases are considered to be disorders of the adaptive immune system. While autoantibodies mediate tissue damage via activation of complement proteins and leukocytes, autoreactive T cells induce damage via delayed-type hypersensitivity (DTH) reaction by secreting primarily T helper (Th)1, (interferon-γ) and Th17 (interleukin-17 family) cytokines. Two major factors have been implicated in the development of autoimmune responses: genetic susceptibility and environmental triggers such as exposure to microbes. Hypothetically, if ∼8% of the U.S. population are affected with some form of autoimmune disease, then it is hard to envision a scenario in which genetic defects exist in all of those affected. Thus, both genetic and environmental factors appear to be inseparable for occurrence of organ-specific autoimmune diseases that also include diseases of the cardiovascular system.

Conversely, auto inflammatory diseases involve direct participation of cells of the innate immune system, importantly neutrophils and macrophages. Cells of the adaptive immune system including major histocompatibility complex (MHC) alleles as well as infectious agents are excluded as the causative factors of auto inflammatory diseases (McGonagle and McDermott, 2006). These disorders could be monogenetic (involvement of a single gene) (Drenth et al., 1999) or polygenetic (involvement of more than one gene) (Doria et al., 2012). For example, mutations in inflammasome-related proteins, particularly NOD-like receptor (NLR) genes, are commonly implicated in auto inflammatory disorders with a phenotype of recurrent inflammation and periodic fever attacks (Zhong et al., 2016). Nevertheless, auto inflammatory diseases can affect the cardiovascular system with a phenotype of myocarditis, pericarditis/inflammatory recurrent acute pericarditis (IRAP), as might occur in sarcoidosis and eosinophilic granulomatosis associated polyangiitis (Dennert et al., 2010a; Bocoum et al., 2011).

**FIGURE 1:**
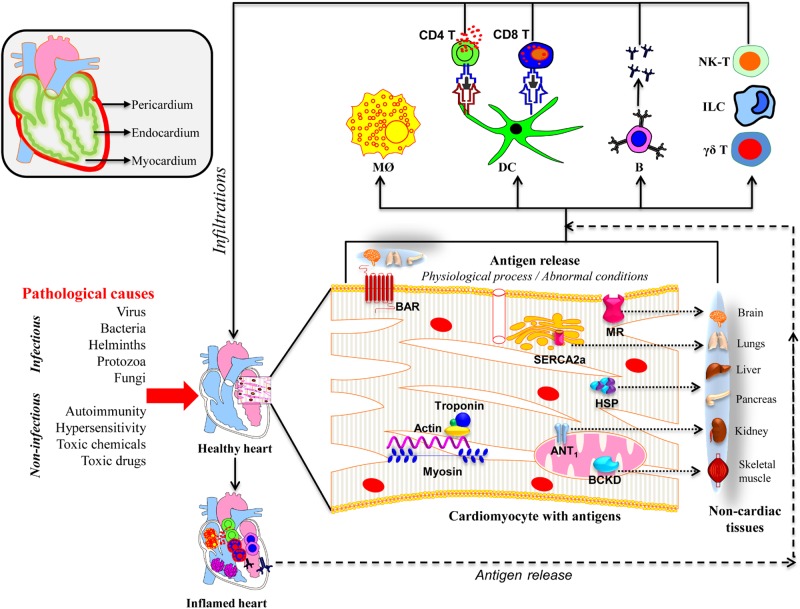
Proposed mechanisms of inflammatory heart disease. All three layers (pericardium, myocardium, and endocardium) individually or together can be affected by various infectious and non-infectious causes. A variety of innate immune cells like neutrophils, macrophages, dendritic cells including γδ T cells, ILCs, and NK-T cells can infiltrate and take part in tissue destruction. The inflammatory damage can also lead to the release of cardiac antigens such as cardiac myosin, actin, cTnI, ANT, BAR, BCKD, SERCA2a, laminin, muscarinic receptor (MR), and heat shock proteins (HSP). The newly released antigens can trigger autoimmune responses by activating T cells and B cells in the draining lymph nodes, which can in turn infiltrate the heart and aggravate inflammation. It is also possible that the dying cardiac myocytes can be engulfed by macrophages as a part of the cleaning process by phagocytosis, and possibly, autophagy, and trigger pathogenic autoimmune responses in genetically susceptible individuals. Alternatively, misfolded or mutated or modified self-proteins as might occur with mitochondrial proteins can be seen by the immune system as foreign, resulting in pathological autoimmune responses. It also is possible that some of the intracellular proteins like HSPs may bear sequences similar to microbial HSPs leading to the generation of cross-reactive immune responses. In all these scenarios, self-antigens can be presented to both CD4 and CD8 T cells, and B cells. They mediate inflammation through, respectively, DTH, cytolysis and complement activation. However, it is to be noted that several putative cardiac antigens can be promiscuously expressed in non-cardiac tissues such as brain, lungs, liver, pancreas, kidney, and skeletal muscle, raising the question whether autoreactive responses generated in response to cardiac damage can also inflict damage in other organs.

In this review, we discuss the mechanistic insights into the development of inflammatory heart disease (IHD), a group of disorders that affect different layers of the heart (Figure 1). These include pericarditis (inflammation of the outer membranous sac), myocarditis (inflammation of the heart muscle), and endocarditis (inflammation of the inner lining of the heart and heart valves and/or coronary arteries) (Fairweather and Rose, 2005; Lu et al., 2015).

### Pericarditis

The pericardium is made up of an outer single-layered fibrous membranous sac, and an inner double-layered serous sac that covers the heart and great blood vessels. Pericarditis, although self-limiting, can be complicated by pericardial effusion or pericardial constriction (Troughton et al., 2004). While, 40–85% of patients affected with pericarditis may have unknown etiologies, recurrences can occur in up to 20–50% of those affected (Brucato et al., 2008; Fancello et al., 2014). Acute pericarditis can be seen in all age groups, but males are more commonly affected than females in the age group of 20–50 years (Ariyarajah and Spodick, 2007). Estimates indicate that 0.1% of people admitted to hospitals with chest pain can have pericarditis (Imazio and Trinchero, 2007). Similarly, 5% of those admitted with chest pain in emergencies unrelated to myocardial infarction might have pericarditis (Imazio et al., 2010; LeWinter, 2014). The disease is self-limiting in most patients (70–90%) and respond well to anti-inflammatory medications like aspirin or non-steroidal anti-inflammatory drugs with colchicine, corticosteroids, and anakinra (IL-1 receptor antagonist) (Imazio et al., 2010). A variety of infectious and non-infectious triggers have been implicated in the causation of pericarditis (Table 1). Microbes frequently associated with pericarditis include bacteria [*Staphylococcus aureus*, *Streptococcus*, *Haemophilus*, *Coxiella burnetti*, and *Mycobacterium tuberculosis* (M. tb)] (Pankuweit et al., 2005; Brucato et al., 2008), viruses [Echovirus, Coxsackievirus B (CVB), parvovirus B19, human herpes virus 6 (HHV6), Epstein-Barr Virus (EBV), human immunodeficiency virus (HIV), and influenza B virus (IBV)] (Brucato et al., 2008). The disease can also arise from co-infections caused by multiple organisms such as *S. aureus* and M. tb in immune-suppressive individuals as might occur in HIV infection imposing a clinical challenge to target specific pathogens for therapy particularly in patients with recurrences (Lamas et al., 2019).

**TABLE 1:** Potential major causes of inflammatory heart disease.

	**Microbes**	**Non-microbes**
Pericarditis	**Viruses**	Systemic lupus erythematosus
	Enteroviruses (CVB and echovirus)	Sjögrens syndrome
	Herpesviruses (EBV and HHV6)	Rheumatoid arthritis
	Adenovirus	Scleroderma
	Parvovirus B19	Familial Mediterranean fever
	Influenza virus	TNFR-associated periodic syndrome
	Hepatitis B and C viruses	Neoplasms
	Human immunodeficiency virus	
	**Bacteria**	
	*Mycobacterium tuberculosis*	
Myocarditis	**Viruses**	
	Enteroviruses (CVB and echovirus)	Inflammatory bowel disease
	Erythroviruses (Parvovirus B19)	Giant cell myocarditis
	Adenovirus	Diabetes mellitus
	Human herpesvirus 6 and 7	Sarcoidosis
	Hepatitis C virus	Systemic lupus erythematosus
	Human immunodeficiency virus	Thyrotoxicosis
	Epstein-Barr virus	Alcohol
	Cytomegalovirus	Doxorubicin
	Influenza virus	*Hypersensitivity*
	**Bacteria**	Sulphonamides
	*Corynebacterium diphtheriae*	Penicillins
	*Staphylococcus aureus*	Digoxin
	*Streptococcus aureus*	
	*Borrelia burgdorferi*	
	*Mycoplasma pneumoniae*	
	*Treponema pallidum*	
	**Fungus**	
	*Aspergillus* species	
	*Candida* species	
	*Coccidioides* species	
	*Cryptococcus* species	
	*Histoplasma* species	
	**Protozoa**	
	*Trypanosoma cruzi*	
	*Babesia* species	
Endocarditis	**Bacteria**	Intravenous drug usage
	*Staphylococcus aureus*	Rheumatoid arthritis
	*Streptococci* species	Systemic lupus erythematosus
	*Enterococci* species	Diabetes mellitus
	*Haemophilus* species	Cancer
	*Aggregatibacter* species	Poor dental health care
	*Cardiobacterium* species	
	*Actinobacillus actinomycetemcomitans*	
	*Eikenella corradens*	
	*Kingella* species	
	**Fungus**	
	*Candida* species	

*CVB, Coxsackie B virus; EBV, Epstein-Barr virus; HHV6, human herpesvirus 6; TNFR, tumor necrosis factor receptor.*

The most alarming complication of acute pericarditis is IRAP, which occurs in 15–30% of patients due to auto inflammatory or autoimmune events (Lazaros et al., 2017). Auto inflammatory reactions in IRAP can result from virus infections such as Echovirus, CVB, and bacteria like M. tb, but the affected individuals should be negative for microbe-specific RNA and antibodies (IgM) to qualify for IRAP-diagnosis (Imazio et al., 2010; Bogdanos et al., 2013; LeWinter, 2014). However, the IRAP patients can carry anti-heart (AHA: myosin heavy chain β and β and myosin light chain-1v isoform) and anti-intercalated-disk autoantibodies, in addition to non-cardiac autoantibodies (Maisch et al., 1991; Latif et al., 1993). Impaired function of the innate immune system resulting in auto inflammatory pericarditis can occur due to mutations in immune response genes. Two such examples include mutations in *MEFV* and *TNFRSF1A* that cause Mediterranean fever and TNF receptor-associated periodic syndrome, respectively (Cantarini et al., 2010; Maggiolini et al., 2011).

As for autoimmune pericarditis, involvement of the pericardium has been reported in systemic autoimmune diseases such as systemic lupus erythematosus (SLE), rheumatoid arthritis (RA), progressive systemic sclerosis, Sjögrens syndrome, and polyarthritis, but the affected patients can remain asymptomatic (Cantarini et al., 2015). Pericardial fluid, and not plasma samples, may contain inflammatory mediators like IL-6, IL-8, and IFN-γ with a preferential detection of anti-myolemma over anti-sarcolemma antibodies, implying that local autoimmune events can occur specific to the heart (Pankuweit et al., 2000). Recent reports indicate that serum carcinoembryonic antigen cell adhesion molecule 1 and MHC class I chain-related protein A can be used as biomarkers and prognostic markers in pericarditis patients, respectively, whereas the appearance of cardiac troponin-T (cTnT) signifies occurrence of acute and recurrent pericarditis (Hamm et al., 1997; Gamaza-Chulián et al., 2014).

### Myocarditis

Myocarditis may involve cardiac myocytes, interstitial, or vascular elements of the heart that can be manifested as perimyocarditis involving the pericardium. Affected patients may show clinical manifestations of disease or may remain asymptomatic, but histopathologic changes can be detected in those affected (Fabre and Sheppard, 2006). Myocarditic hearts can contain variable numbers of lymphocytes and macrophages, but antibody-mediated injury also can be expected (Cooper, 2009; Schultz et al., 2009). The disease is generally regarded as an underdiagnosed cause of acute heart failure, and sudden death or dilated cardiomyopathy (DCM) can be expected in young adults (Drory et al., 1991). The annual global prevalence of myocarditis has been estimated to be ∼22 cases per 100,000 patients (Drory et al., 1991; Roth et al., 2015), and 1–5% of acute viral infections may have myocardial involvement (Fairweather and Rose, 2005). Furthermore, myocarditis is relatively more common in young men than their female counterparts, indicating that sex hormones can influence the disease outcome (Kytö et al., 2013). While virus-induced myocarditis in neonates and children may lead to fulminant myocarditis, lymphocytic or giant cell myocarditis is generally noted in the median age group of 42–43 years (Rose, 2016). Approximately 10–20% of those affected with acute myocarditis as young adults develop chronic myocarditis, DCM, and congestive heart failure. Approximately half of these patients undergo heart transplantation due to the lack of effective treatment options (Caforio et al., 2010; Rose, 2016).

Myocarditis can occur in association with a wide spectrum of infectious agents, systemic diseases, and hypersensitivity to drugs and toxins (Table 1). While viral infections caused by enteroviruses like CVB, adenoviruses, parvovirus B19, CMV, EBV, HIV, hepatitis C virus, and influenza virus are commonly suspected as causes of myocarditis in the developed world (Pollack et al., 2015), rheumatic carditis/diphtheria caused by *Streptococcus aureus* and Chagas disease caused by *Trypanosoma cruzi* are implicated in developing countries (Anez et al., 1999; Grumbach et al., 1999; Arbustini et al., 2000; Nolte et al., 2000; Boruah et al., 2010). More recently, it has been noted that patients receiving checkpoint inhibitors for tumors can develop autoimmune myocarditis, raising the question whether anti-tumor T cells may recognize cardiac antigens by cross-reactivity with microbial antigens (Brumbaugh et al., 2019; Martin Huertas et al., 2019). In support of this proposition, it was recently shown translationally that commensal bacteria can promote inflammatory cardiomyopathy by elevating *Bacteroides-*specific CD4 T cell and B cell responses in human myocarditis patients (Gil-Cruz et al., 2019). This observation was also complemented in the mouse model, where cardiac myosin-specific Th17 cells could be activated by cross-reactive mimic peptides from *Bacteroides thetaiotaomicron*. These findings suggest that the microbiome could predispose genetically susceptible individuals undergoing checkpoint inhibitors to develop myocarditis, leading to the suggestion that they can potentially be treated with antibiotics (Gil-Cruz et al., 2019).

In North America, myocarditis/DCM has been frequently associated with enterovirus infections like CVB, and approximately 50% of DCM patients can carry CVB-reactive antibodies, and viral RNA can be detected in 70% of them (Baboonian and McKenna, 2003; Fujinami et al., 2006). However, other viruses namely, parvovirus B19 and HHV-6 are now being increasingly reported in association with myocarditis/DCM (Andreoletti et al., 2009; Pollack et al., 2015). The CVB infection is generally used to understand how viral infections can trigger autoimmune responses. CVB pathogenesis involves both virus-induced (direct) and host-induced (indirect) mechanisms (Cihakova and Rose, 2008). Because it is a cardiotropic and lytic virus, CVB can directly lyse cardiomyocytes during viral replication. Experimentally, CVB infection in mice can have two phases in continuum: acute myocarditis (viral) up to day 14–18 and chronic myocarditis (non-viral) beyond 18 days (Rose et al., 1986). During the initial stages of CVB infection, activation of innate immune cells results in the production of the pro-inflammatory cytokines IL-1, IL-6, TNF-β, soluble IL-1R-like 1 also called, sST2 and IFNs (Fairweather et al., 2003; Nishimura et al., 2018). Such proteins can potentially be used as markers of myocarditis. For example, it has been recently shown that men with myocarditis had higher levels of sST2 than women. This finding was later confirmed experimentally, where gonadectomy with testosterone, but not estradiol replacement, led to increased levels of sST2 in male mice with myocarditis (Coronado et al., 2019). These data also add credence to the relevance of experimental models to human diseases. As to adaptive immune response, Th cells appear to play a decisive role in the induction of chronic myocarditis/DCM, but involve complex mechanisms. While, the Th1 cells can protect acute myocarditis and prevent viral replication (Fairweather et al., 2004; Fairweather et al., 2005), Th2 cells, although they have the ability to attenuate acute myocarditis via anti-inflammatory cytokines and Treg cells (Frisancho-Kiss et al., 2007; Liu and Huber, 2011), can induce acute cardiac remodeling leading to chronic myocarditis/DCM (Abston et al., 2012). Conversely, Th17 cells contribute to the development of both acute myocarditis (but without affecting viral replication), as well as cardiac remodeling/DCM events and heart failure (Fairweather et al., 2012) suggesting that the disease course of viral myocarditis may be dictated by nature and the amount of Th cytokines being produced. Nonetheless, these findings raise a question as to the mechanisms of generation of pathogenic cardiac-reactive T cells, should they be antigen-specific.

One mechanism is molecular mimicry, which involves the generation of cross-reactive immune responses against self-antigens. In fact, sequence similarities of up to 40% have been detected between CVB viral protein_1_ (VP_1_), streptococcal M protein, and cardiac myosin; synthetic peptides containing these sequences block monoclonal antibody (mAb) reactivity to streptococcal M protein (Cunningham et al., 1992). Furthermore, murine anti-streptococcal mAbs that cross-react with streptococcal M protein and human cardiac myosin were found to neutralize CVB viruses (CVB3 and CVB4) and poliovirus type 1 (Cunningham et al., 1992). Additionally, T lymphocytes from CVB-infected animals can respond to peptides from streptococcal M protein (Huber et al., 1994). Similarly, the N-terminal region four peptide from streptococcus could induce IHD in MRL/++ mice, whereas induction of anergy to this peptide protects mice from developing CVB-induced myocarditis (Huber and Cunningham, 1996).

Another mechanism is epitope spreading which may occur as a result of release of self-antigens primarily by viral damage, leading to induction of autoimmunity secondarily (Fujinami et al., 2006). In addressing this phenomenon, we had demonstrated that A/J mice infected with CVB produce Myhc-α 334-352-specific CD4 T cell responses that are pathogenic in nature (Gangaplara et al., 2012). Using MHC class II dextramers specific to Myhc 334–352, we showed that Myhc-specific, CD4 T cells can infiltrate into the hearts of animals infected with CVB during the post infectious phase of CVB infection (Gangaplara et al., 2012). By expanding this observation, we may be able to determine whether autoreactive T cells specific to multiple cardiac antigens could contribute to CVB pathogenesis. This effort, however, requires identification of myocarditogenic epitopes for other putative cardiac antigens implicated in myocarditis/DCM patients (Figure 1).

### Endocarditis

Endocarditis can also be caused by infective or non-infective agents (Table 1), that have a male preponderance (Aksoy et al., 2007). Incidence of infective endocarditis in developed countries is 3–10 cases per 100,000 people, and the disease prevalence is expected to be high in developing countries (Duval et al., 2012; Selton-Suty et al., 2012). Endocarditic lesions, primarily called vegetation, consist of microorganisms, inflammatory cells, platelets, and fibrin. Microbes such as *S. aureus*, *Streptococcus* species, *Enterococcus* species, and HACEK Gram-negative bacteria (*Haemophilus* species, *Actinobacillus actinomycetemcomitans*, *Cardiobacterium hominis*, *Eikenella corrodens* and *Kingella* species) account for more than 90% of endocarditis cases that may have a nosocomial or odontogenic origin (Table 1; Murdoch et al., 2009; Raza et al., 2010; Ashrafi et al., 2012; Selton-Suty et al., 2012). However, highest mortalities occur with *S. aureus* and the non-HACEK group pathogens such as *Candida albicans* (Ashrafi et al., 2012). Additionally, infective endocarditis also can occur in intravenous drug abusers or in association with HIV infection, diabetes mellitus, rheumatoid arthritis, Sjögrens syndrome and cancer (Miro et al., 2002; Filizcan et al., 2004; Sughimoto et al., 2006; Yoshioka et al., 2018; Sanaiha et al., 2019; Table 1). These observations suggest that multiple pathomechanisms may contribute to the development of endocarditis.

Damage to the endocardium can result from valve sclerosis, rheumatic valvulitis, or direct colonization/vegetation by bacteria like *S. aureus* and *Streptococcus* species (Cahill and Prendergast, 2016). The main sequelae of endocarditis include damage to the heart valve and endothelial scarring, leading to congestive heart failure as might occur in autoimmune rheumatic diseases. Endocarditis resulting from *Streptococcus* species., has been used as a prototypic disease model to address how cross-reactive autoimmune responses can lead to heart dysfunction (Krisher and Cunningham, 1985; Cunningham et al., 1986). It has been shown that mAbs specific to Streptococcus M protein or the group A streptococcal epitope *N*-acetyl-βD-glucosamine can cross-react with cardiac myosin and laminin (Krisher and Cunningham, 1985; Galvin et al., 2000). The finding that anti-streptococcal antibodies cross-react with human and canine cardiac sarcolemma proteins but not kidney or skeletal muscle, suggests that sarcoplasmic antigens can be targeted in autoimmune endocarditis (Dale and Beachey, 1982; Cunningham et al., 1986).

Animal models have been developed to study the immune pathology of endocarditis and valvular inflammation. Intraperitoneal injection of cell lysate from group A streptococci into Swiss Webster mice results in aortic and mitral valve stenosis, chronic inflammation, and scarring that mimic human chronic rheumatic carditis (Cromartile and Craddock, 1966; Cunningham et al., 1984). In the Lewis rat model, immunization with streptococcal M protein leads to the induction of cross-reactive T cells that are pathogenic in nature (Quinn et al., 2001). Similarly, Lewis rats immunized with M protein can develop valvulitis in mitral valves, but with minimal myocardial involvement (Quinn et al., 2001). Expectedly, lymph node cells obtained from immunized animals respond to human cardiac myosin, but not human and rabbit skeletal myosin and actin, although both human cardiac and skeletal myosin share extensive sequence identity (Quinn et al., 2001). However, it is interesting that, in spite of protein sequence identity between streptococcal M proteins and cardiac myosin, myocardial involvement was noted to be minimal relative to valvulitis in the immunized Lewis rats. Likewise, laminin present in the valves has been shown to cross-react with both anti-streptococcal and myosin antibodies, and cross-reactive T cells generated by immunization with laminin can induce valvulitis without significant involvement of the myocardium (Quinn et al., 2001).

Endocarditis can be seen in patients with RA, SLE, and rheumatic fever, in which both joint and cardiovascular systems could be affected. RA patients can also carry cardiac-specific antibodies (Breed and Binstadt, 2015). To study the immune-pathogenic mechanism of inflammatory arthritis, the K/BxN TCR transgenic mouse model has been developed (Monach et al., 2008). The transgenic mice express TCR specific to glucose-6-phosphate isomerase (GPI), which plays a key role in catalyzing the interconversion of glucose-6-phosphate to fructose-6-phosphate (Chang and Wei, 2011). GPI-primed B cells produce autoantibodies that can induce arthritis (Monach et al., 2007). Recent studies suggest that transgenic mice can develop both arthritis and endocarditis, but through two independent pathways. While complement C5 protein is critical for development of arthritis, but not endocarditis, occurrence of endocarditis, but not arthritis, requires the participation of FcRγ (Binstadt et al., 2009). Adoptive transfer of serum from transgenic mice into naïve animals led to the development of arthritis that was devoid of endocarditis, implying that arthritis is an antibody-mediated disease (Maccioni et al., 2002). Conversely, animals depleted of CD4^+^ T cells fail to develop endocarditis, but arthritis remains unaffected, suggesting that T cells play a critical role in the development of endocarditis (Binstadt et al., 2009). Such disease models may be helpful in studying the immune mechanisms of rheumatic diseases that involve both synovial joints and/or the endocardium.

## Discussion

As described above, etiologically, IHD can be triggered by a wide range of microbes, therefore posing a clinical challenge, that necessitates identification of triggers to initiate pathogen-specific therapies. Such avenues are clinically complicated, since affected patients can have co-infections caused by microbes belonging to different classes. For example, HIV patients can develop pericarditis in association with *S. aureus* and M. tb infections (Lamas et al., 2019). Similarly, myocarditis can result from co-infection with HHV-6 and parvovirus B19 (Rohayem et al., 2001). Likewise, some infections like EBV and parvovirus B19, may occur as a result of their reactivation, respectively, in CVB3 and hepatitis virus, and HHV-6 infections (Bock et al., 2014; Atti et al., 2017). Such scenarios are likely because up to 95% of the United States population is affected with EBV infection (Atti et al., 2017). Furthermore, tissue destruction may not necessarily result directly from cardiotropic pathogens like CVB3 and adenovirus. For example, parvovirus B19 targeting endothelial cells can induce myocarditis via endothelial dysfunction and initiate inflammation (Bock et al., 2010).

Nevetheless, following infection-tiggered IHD, the immune system in immune-competent individuals is expected to repair the damaged tissue with or without successful clearance of primary offenders. But such an ability of the immune system to self-cure is relatively limited for cardiac cells, especially adult cardiac myocytes because of their limited regenerative capacity (Kikuchi and Poss, 2012). This scenario can be complicated by the potential occurrence of secondary damage resulting from autoimmune responses in genetically susceptible individuals. In addressing this phenomenon, we have noted that CVB3 infection can lead to the generation of cardiac myosin-specific T cells that can contribute to post-infectious myocarditis in the absence of detectable virions in infected animals (Gangaplara et al., 2012). Based on this observation, we envision that cardiac-reactive T cells specific for multiple autoantigens could be generated in infections caused by cardiotropic pathogens that may have implications in diagnosis and therapy of IHD.

Diagnostically, it is extremely challenging to identify the causative agents for IHD with certainty, especially if polymicrobes (co-infections) are involved. Additionally, one or more manifestions of IHD (pericarditis, myocarditis, or endocarditis) can potentially occur concurrently in affected patients. Nonetheless, it may be possible to develop diagnostic panels for commonly found pathogens like viruses and bacteria. These may include PCR-based molecular markers or antibody assays with the challenge that clinical specimens, especially endomycardial biopsies, need to be collected in a timely fashion to make a reliable diagnosis. This is especially important in chronically affected individuals with no overt clinical signs suggestive of known microbial infections. Such an approach, if supported by routinely used serum biomarkers namely, cardiac troponin I and T, creatinine kinase-MB, C-reactive protein, myoglobin, natriuetic peptides, and sST2 (Biomarkers, 2014; Nishimura et al., 2018; Coronado et al., 2019; Kottwitz et al., 2020) may have diagnostic value. A limitation of this approach is that the offending agents would be gone by that time, except being suspects, which may not alter treatment approaches to be followed clinically.

Thus, the complex nature of IHD is therapeutically challenging in the medical management of affected individuals. Although, various clinical trials reported thus far have yielded mixed successes, two major approaches seem to be encouraging. These include immune therapies (administration of intravenous immunoglobulins or immunoglobulin adsorption), and anti-viral agents. The former approach appeared to be effective in patients with giant cell myocarditis and sarcoidosis who are negative for viruses (Pollack et al., 2015). Conversely, this approach offered no advantage in those positive for viruses (Pollack et al., 2015). The latter group of patients, however, can potentially be treated with anti-viral agents as reported in parvovirus B19 (peramivir) and influenza (acyclovir) infections, with or without immune suppressive therapy, respectively (Dennert et al., 2010b; Baik et al., 2015). Although such outcomes are rarely reported, the therapeutic value of anti-viral compounds can be validated only in large clinical trials that involve careful recruitment of patient populations and appropriate controls. Finally, as to autoimmunity, use of general immune suppressants potentially elimimate most naïve lymphocytes that can seriously impair a host’s defense mechanisms against microbes. Thus, immune strategies are expected to target disease-inducing, autoreactive T cells or B cells by sparing naïve repertoires. To this end, various selective immune suppressants have been investigated that may hold promise in the future.

## Author Contributions

All authors contributed to the synthesis of literature and writing the manuscript.

## Conflict of Interest

BY was employed by Bristol-Myers Squibb. The remaining authors declare that the research was conducted in the absence of any commercial or financial relationships that could be construed as a potential conflict of interest.

## Abbreviations

AHA: anti-heart antibodies
ANT: adenine nucleotide translocator
BAR: beta adrenergic receptor
BCKD: branched chain ketoacid dehydrogenase
CMV: cytomegalovirus
cTnI: cardiac troponin I
cTnT: cardiac troponin T
CVB: Coxsackievirus B
DCM: dilated cardiomyopathy
DTH: delayed-type hypersensitivity
EBV: Epstein-Barr virus
GPI: glycosyl phosphatidyl inositol
HHV: human herpesvirus
HIV: human immunodeficiency virus
HSPs: heat shock proteins
IBV: influenza B virus
IFN: interferon
IHD: inflammatory heart disease
IL: interleukin
ILCs: innate lymphoid cells
IRAP: inflammatory recurrent acute pericarditis
mAb: monoclonal antibody
MHC: major histocompatibility complex
M. tb: *Mycobacterium tuberculosis*
NK-Ts: natural killer-T cells
NLR: NOD-like receptor
PCR: polymerase chain reaction
RA: rheumatoid arthritis
SERCA2a: sarcoplasmic/endoplasmic reticulum Ca^2+^ ATPase 2a
SLE: systemic lupus erythematosus
TCR: T cell receptor
Th: T helper
TNF: tumor necrosis factor.
